# Knockdown of the stem cell marker Musashi-1 inhibits endometrial cancer growth and sensitizes cells to radiation

**DOI:** 10.1186/s13287-022-02891-3

**Published:** 2022-05-26

**Authors:** Isabel Falke, Fabian M. Troschel, Heike Palenta, Maria T. Löblein, Kathrin Brüggemann, Katrin Borrmann, Hans Theodor Eich, Martin Götte, Burkhard Greve

**Affiliations:** 1 Department of Radiation Oncology, University Hospital Münster, Albert-Schweitzer-Campus 1, 48149 Münster, Germany; 2 Department of Gynecology and Obstetrics, University Hospital Münster, 48149 Münster, Germany

**Keywords:** Musashi-1, Cancer stem cell, Endometrial cancer, Proliferation, Radioresistance

## Abstract

**Background:**

Endometrial carcinoma is the most common gynecological cancer in Europe. Musashi-1 is known to be a key regulator of endometrial cancer stem cells and a negative prognostic marker. In the present study, we aimed to understand growth and gene expression patterns in endometrial carcinoma after Musashi-1 knockdown in vitro and in vivo. Changes in therapeutic resistance were also assessed.

**Methods:**

First, we performed analyses to understand Musashi-1 expression patterns using The Cancer Genome Atlas database. We then proceeded to assess effects of small interfering RNA-based Musashi-1 targeting in two endometrial carcinoma cell lines, Ishikawa and KLE. After quantifying baseline changes in cell metabolism, we used MTT tests to assess chemotherapy effects and colony formation assays to understand changes in radioresistance. For mechanistic study, we used quantitative polymerase chain reaction (qPCR) and western blotting of key Musashi-1 target genes and compared results to primary tissue database studies. Finally, xenograft experiments in a mouse model helped understand in vivo effects of Musashi-1 knockdown.

**Results:**

Musashi-1 is aberrantly expressed in primary tumor tissues. In vitro, silencing of Musashi-1 resulted in a strong decline in cell proliferation and radioresistance, while chemoresistance remained unchanged. Loss of Musashi-1 led to downregulation of telomerase, DNA-dependent protein kinase, the Notch pathway and overexpression of cyclin-dependent kinase inhibitor p21, the latter of which we identified as a key mediator of Msi-1 knockdown-related anti-proliferative signaling. In vivo, the anti-proliferative effect was confirmed, with Msi-1 knockdown tumors being about 40% reduced in size.

**Conclusions:**

Musashi-1 knockdown resulted in a strong decrease in endometrial cancer proliferation and a loss of radioresistance, suggesting therapeutic potential.

**Supplementary Information:**

The online version contains supplementary material available at 10.1186/s13287-022-02891-3.

## Background

Endometrial carcinoma (EC) shows a varying incidence worldwide, but is the most common gynecologic malignancy in developed countries [1, 2]. Given early onset of symptoms, most tumors are limited to the uterus at time of diagnosis. In Europe, overall 5-year disease-specific survival is 76% [2], but is reduced to 17% in patients with distant metastases [3, 4].

While most patients are treated with hysterectomy and bilateral salpingo-oophorectomy, patients with a high risk of recurrence or those who have been diagnosed with advanced disease may also undergo radio- and/or chemotherapy. Definitive radiotherapy is indicated for primary tumors when surgery is contra-indicated [5].

New molecular markers are currently investigated to guide adjuvant therapeutical decisions [5]. Histologically, the most common EC subtype is the endometrioid type (type 1 EC), with a positive hormone receptor status, indicating estrogen dependency. Type 2 EC is less differentiated, more aggressive, and associated with a worse prognosis [6]. Here, we chose to investigate Ishikawa, a type 1 EC cell line, while a second cell line, KLE, was representative of type 2 EC [7].

Musashi-1 (Msi-1) is a small, 39 kDa intracellular RNA-binding protein implicated in stem cell renewal, differentiation and maintenance of pluripotency [8]. Msi-1 is a bifunctional translational regulator either repressing [9, 10] or activating its target genes [11]. Msi-1 was found to be overexpressed and of prognostic relevance in multiple malignancies [12–15].

Previous studies by our group showed Msi-1 to be overexpressed in EC, especially in cancer stem cells (CSC) and also linked Msi-1 to Notch-1 expression [16]. We additionally found Msi-1 knockdown to increase apoptosis and to modulate cell cycle progression [17]. Finally, Msi-1 has recently been shown to be a negative prognostic marker in EC [18].

While some mechanistic interplay has been uncovered, the therapeutic potential of Msi-1 targeting with regard to cell viability and therapy resistance remains largely unknown. Hence, the aim of the present study was to identify potential therapeutic effects of Msi-1 knockdown on proliferation as well as irradiation and chemotherapy treatment.

## Methods

### Cell culture

Ishikawa cells were maintained in MEM Eagle medium supplemented with 10% fetal calf serum (FCS), 1% Penicillin/Streptomycin solution, 1% Non-Essential Amino Acids (all PAN Biotech, Aidenbach, Germany) and 25 mM Hepes (Roth, Karlsruhe, Germany).

KLE cells were cultured in DMEM/F12 medium supplemented with 10% FCS, 1% Penicillin/Streptomycin solution (all PAN Biotech) and 25 mM Hepes (Roth). Both cell lines were grown at 37 °C in a 5% CO_2_ containing, H_2_O saturated incubator.

### Musashi-1 knockdown in vitro

Transient short interfering RNA (siRNA)-mediated knockdown was performed using lipofectamine RNAiMax (Invitrogen, Thermofisher Scientific, Waltham, MA, USA) per the manufacturer’s instructions. We used two different, independent experimental siRNA-based approaches to verify results and eliminate the risk of off-target effects: (1) We combined two Msi-1 siRNAs at equimolar concentrations of 2.5 nM (ambion by life technologies, Thermofisher Scientific, Additional file 1: Table S1). SiRNAs were combined to target multiple different exons of the Msi-1 mRNA and to reduce off-target effects. Meanwhile, control cells were treated with negative control siRNA (Thermofisher Scientific). (2) Key Western Blot targets and colony formation experiments were independently verified using a Musashi-1 siPOOL (siTOOLs Biotech, Planegg, Germany) containing a mix of 30 different Msi-1 specific siRNAs. This approach has also been developed to eliminate off-target effects [19]. A concentration of 2.5 nM was used. Lipofectamine RNAiMax (Invitrogen) was added for transfection, and control cells were treated with a control siPOOL (siTOOLs Biotech).

Cells were incubated for 24 h with transfection reagent. Afterward, standard medium was used.

### Double knockdown of Musashi-1 and p21 in vitro

To assess the relevance of the Msi-1-p21 axis, we performed knockdown of both p21 and Msi-1, using specific siPOOLs (siTOOLs Biotech). Again, lipofectamine and siPOOL concentrations of 2.5 nM of p21, Msi-1 and negative control were used. Cells were incubated for 24 h. Afterward, cells were resuspended in standard medium. Detailed information on siRNAs/siPOOLs can be found in Additional file 1: Table S1.

### Reverse transcription-quantitative polymerase chain reaction (RT-qPCR)

Total RNA was isolated from cell lines 72 h after transfection using RNeasy® Mini Kit (Qiagen, Venlo, The Netherlands) and transcribed using the High Capacity cDNA Reverse Transcription Kit (Applied Biosystems, Thermofisher Scientific). Expression levels were measured using TaqMan® Gene Expression Master Mix (Applied Biosystems, Thermofisher Scientific) with 18S serving as endogenous control. Real-time PCRs were performed on a Rotor-Gene Q machine (Qiagen) in triplicates for each experiment and analyzed using the comparative cycle threshold method (2^−∆∆Ct^) [20]. Materials are listed in Additional file 1: Table S2.

### Western Blot analysis

72 h after transfection, cells were lysed and protein was quantified using Bradford reagent (Sigma-Aldrich, Merck Life Science). 35 µg of protein per lane was separated using electrophoresis on 4 to 20% Mini-Protean TGX Gels (Bio-Rad, Hercules, CA, USA) in Mini Protean Tetra Systems (Bio-Rad) at 25 mA and transferred overnight to a nitrocellulose membrane at 10 V (Whatman Protran, Washington, USA). Membranes were probed with primary antibodies overnight at 4 °C followed by incubation with secondary antibodies for 2 h. Pierce ECLT Western Blotting Substrate (Thermofisher Scientific) and X-ray film developer Curix 60 (Agfa, Mortsel, Belgium) were used for visualization. Quantification was performed with ImageJ Software (National Institutes of Health), normalizing to α-tubulin. Antibodies are listed in Additional file 1: Table S3.

### MTT assay and paclitaxel treatment

24 h after transfection, predefined numbers of cells were seeded into 96-well plates in triplicates. After 24 h, medium was replaced by either normal medium, solvent (DMSO) or Paclitaxel at varying concentrations in complete medium and cells were incubated for 96 h. After adding MTT ((3-(4,5-dimethylthiazol-2-yl)-2,5-diphenyltetrazolium bromide) 5 mg/ml solution (Sigma-Aldrich, Merck Life Science, Burlington, MA, USA), cells were returned to the incubator for 4 h. The reaction was stopped using 10% SDS and 50% N,N-Dimethylformamide in pH 4,7 (HCl), and cells were incubated at room temperature in the dark. 20 h later, colorimetric density was measured at 570 nM using Bio-Rad iMark Microplate Reader and MPM 6 program (Bio-Rad). Sample values were normalized to their DMSO control.

### Colony formation assay

24 h after transfection, cells were irradiated with doses of 2, 4 and 6 Gy using a TrueBeam linear accelerator (Varian, Palo Alto, CA, USA). After harvesting of irradiated and unirradiated samples, predefined numbers of cells were seeded in medium supplemented with 20% FCS in 6-well plates in triplicates per condition and incubated for 10 days (Ishikawa Msi-1 knockdown), 12 days (Ishikawa Msi-1/p21 knockdown), or 20 days (KLE Msi-1 and Msi-1/p21 knockdown). Colonies, confluent groups of more than 20 cells, were counted using an Olympus CKX41 microscope (Olympus, Shinjuku, Tokyo, Japan). Analyses were performed as described previously [21] with plating efficiency defined as number of colonies/number of seeded cells and survival fractions defined as plating efficiency after irradiation/plating efficiency of unirradiated cells.

### Musashi-1 mRNA expression after irradiation

24 h after irradiation with 2 Gy, wild-type cells were harvested and prepared for RT-qPCR as described above. Msi-1 mRNA expression of irradiated cells was then compared to unirradiated controls.

### In silico analysis

We performed in silico analyses of The Cancer Genome Atlas (TCGA) data (546 primary tumor and 35 normal endometrium samples) by using the University of Alabama Cancer Database (UALCAN) web resource [22]. Supplementary analyses were performed using proteomic profiles via the National Cancer Institute’s Clinical Proteomic Tumor Analysis Consortium (CPTAC) Confirmatory/Discovery cohort that was integrated in the UALCAN web portal [23].

For gene expression, TCGA Endometrial Cancer gene expression samples (ID: TCGA-UCEC.htseq_fpkm-uq) were used. Data were downloaded from the University of California Santa Cruz “Xena” tool [24]. RNAseq expression level read counts were produced by HT-Seq and normalized by the fragments per kilobase of transcript per million mapped reads upper quartile (FPKM-UQ) method [25]. Spearman correlations were used for analyses.

### In vivo experiments

Experiments with 8-week-old female nude mice (Charles River Laboratories, Sulzfeld, Germany) were approved by the “Landesamt für Natur, Umwelt und Verbraucherschutz Nordrhein-Westfalen” (Reference number 9.93.2.10.36.07.248). Initially, 1 × 10^7^ Ishikawa cells were injected into the subcutis of the dorsolateral limbs of 30 mice. Knockdown was performed using 40 nM of two Msi-1 targeting siRNAs or negative control siRNA (all Applied Biosystems, Thermofisher Scientific, Additional file 1: Table S1) and DharmaFECT (Dharmacon, Thermofisher Scientific) reagent. Transfection reagent was injected every 3 days into the tumor cell containing area to ensure a persistent knockdown.

Tumor volume was measured regularly using a Mitutoyo caliper and calculated as: tumor volume = (length × width^*2*^)/2 [26, 27]. Mice were killed after 38 days. Xenograft tumors were surgically removed and cut in half.

One part was prepared for RT-qPCR using Basic-rna-OLS-Kit (OLS Omni Life Science, Bremen, Germany) and first strand cDNA synthesis Kit (Fermentas, St. Leon-Rot, Germany) as described by the manufacturer. If feasible, two PCRs per tumor were performed as described above.

The other part was fixed in formalin and embedded in paraffin to prepare microsections for immunohistochemical analyses.

For Msi-1 staining, we used the DAKO KIT System according to the manufacturer’s instructions. Primary monoclonal mouse-anti-Msi-1 antibody (R&D systems, Thermofisher Scientific) was applied overnight at 4 °C. After three washes, primary antibodies were detected using anti-mouse EnVision® systems (Dako) and NovaRed® substrate, according to the manufacturer. Sections were counterstained with Mayers haemalum (Merck, Darmstadt, Germany) and embedded in Kaiser’s glycerol gelatin (Merck).

To investigate apoptosis, we used the In Situ cell Death Detection Kit, TMR red (Roche, Merck, Burlington, MA, USA) and incubated cells with the terminal deoxynucleotidyl transferase-mediated dUTP nick end labeling reaction mixture for 1 h at 37 °C.

Mitosis was evaluated by staining sections with Anti-Phospho Histone-H3 (Thr11) primary antibody (Cell Signaling, Danvers, USA) overnight and secondary antibody Alexa Fluor 488 (Invitrogen, Thermofisher Scientific) for 1 h at 37 °C. Sections were coated with VECTASHIELD Mounting Medium containing 4´,6-diamindino-2-phenylindole (DAPI) (Vector Laboratories, Burlingame, CA, USA) to stain nuclear DNA.

Apoptotic cells appeared red, mitotic cells green, and nuclei appeared blue when visualized on a digital laser scan microscope (Nikon ECLIPSE 90i). Software was NIS-Elements AR4.00.007 64bit (Nikon, Chiyoda, Japan). To determine the mitotic cells per mm^2^ and dead cells per mm^2^, five representative areas of the different tumor microsections were counted and mean value was calculated.

### Statistical analysis

All in vitro studies were conducted at least three times in independent experiments if not otherwise stated (e.g., some validation experiments using siPOOLs were completed in independent duplicates given that independent triplicates had previously been performed for the standard approach with two siRNAs—in this setting, siPOOL experiments were merely confirmatory). In vivo experiments were conducted in 15 mice per group. Fold changes are presented as mean values ± standard error of the mean (s.e.m). A p value equal to or smaller than 0.05 in a Student’s t test was considered statistically significant. Results were visualized using GraphPad Prism software (version 8, San Diego, CA, USA).

## Results

### Musashi-1 expression is upregulated in endometrial carcinoma

**Fig. 1:**
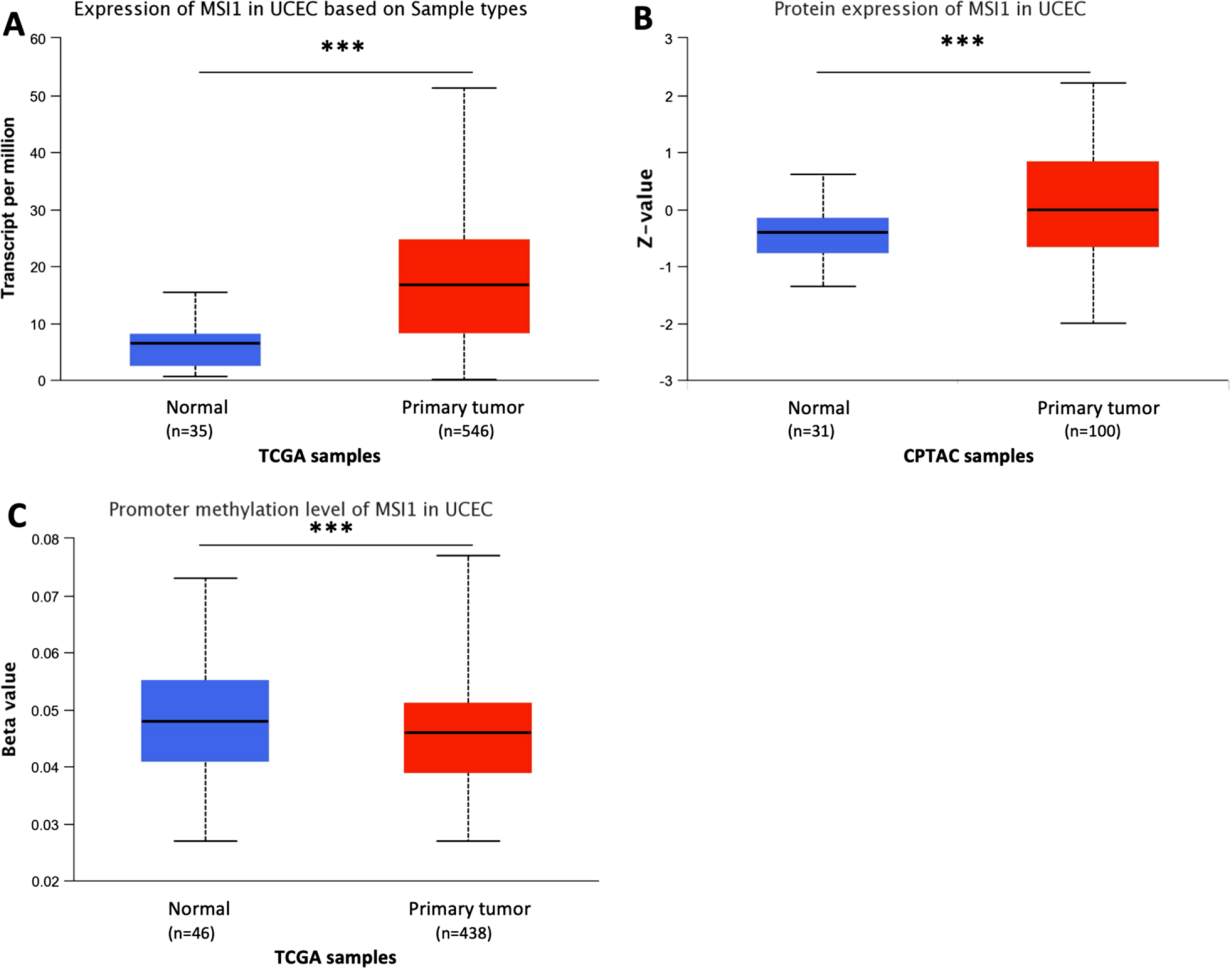
Musashi-1 expression is upregulated in endometrial carcinoma patients, while promoter methylation levels are decreased. **A**, **B** Increased Musashi-1 expression in The Cancer Genome Atlas (TCGA, *p* < 0.001, **A**) and the Clinical Proteomic Tumor Analysis Consortium (CPTAC, *p* < 0.001, **B**) dataset. **C** Decreased promoter methylation levels of Musashi-1 in primary tumor compared to normal endometrium samples (TCGA, *p* < 0.001)

Previous studies in small patient cohorts pointed to a high Msi-1 expression in endometrial cancer patients [16, 18]. Leveraging the availability of TCGA as a large-scale dataset, we confirmed these findings in more than 500 patients (Fig. 1A). Median Msi-1 expression for normal endometrium samples was 6.472 transcripts per million (tpm) compared to 16.813 tpm in primary tumor samples (*p* < 0.001). Similar results were found in the CPTAC dataset in 100 tumor samples (*p* < 0.001, Fig. 1B).

DNA hypomethylation promotes gene and therefore protein expression and is important for early stages of cancer development [28]. Msi-1 promoter methylation analyses in TCGA dataset revealed reduced levels in primary tumor samples compared to normal endometrium tissue (Fig. 1C, *p* < 0.001), supporting increased Msi-1 protein expression in tumor samples.

These findings demonstrating abundant expression in EC informed our subsequent experiments to artificially downregulate Msi-1 in EC cells.

### Musashi-1 knockdown leads to decreased cell metabolism and colony formation in vitro

We performed siRNA-based Msi-1 knockdown in KLE and Ishikawa cells, first confirming knockdown success via qPCR and Western Blotting for two experimental approaches: (1) Use of two siRNAs was the standard approach (Additional file 1: Fig. S1) and (2) use of a siPOOL for additional verification of key experiments (Additional file 1: Fig. S2, blots in Additional file 1: Fig. S3).

**Fig. 2:**
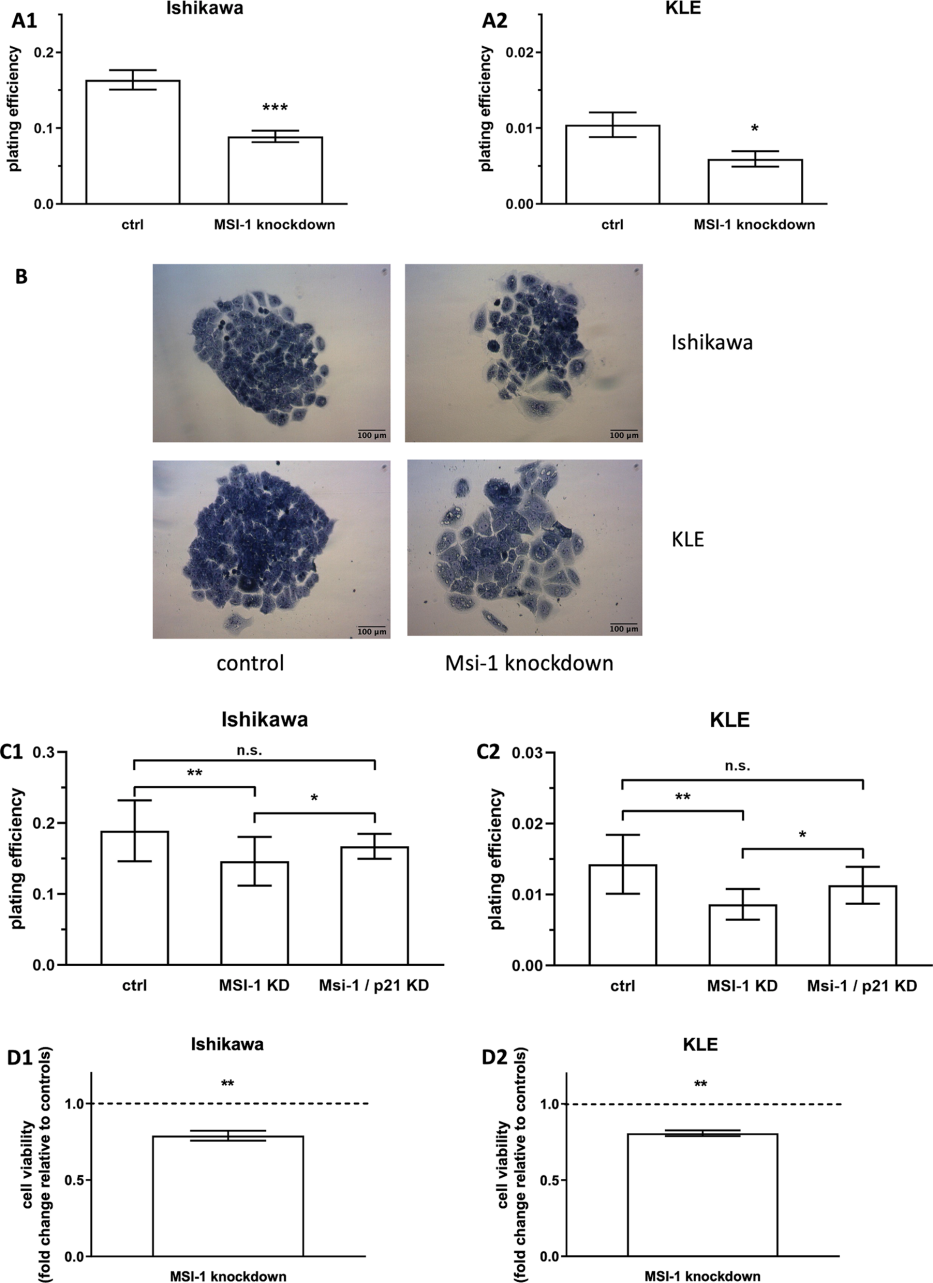
Musashi-1 knockdown leads to decreased cell proliferation and metabolism in vitro. **A** Plating efficiency of Musashi-1 depleted cells (using two siRNAs for the knockdown) compared to control cells in Ishikawa (A1, *p* < 0.001, *n* = 14) and KLE cell line (A2, *p* = 0.027, *n* = 20). **B** Representative images. **C** Decreased colony formation after Musashi-1 knockdown (Msi-1 KD) is partly restored after additional knockdown of p21 (Msi-1/p21 KD) in siPOOL experiments. (**C1** Ishikawa: mean plating efficiency 0.189 for ctrl, 0.146 for Msi-1 KD and 0.167 for Msi-1/p21 KD, *p* = 0.003 for Msi-1 KD compared to controls, *p* = 0.041 for Msi-1 KD compared to Msi-1/p21 KD and *p* = 0.076 for Msi-1/p21 KD compared to controls; **C2** KLE: mean plating efficiency 0.014 for ctrl, 0.008 for Msi-1 KD and 0.011 for Msi-1/p21 KD, *p* = 0.003 for Msi-1 KD compared to controls, *p* = 0.036 for Msi-1 KD compared to Msi-1/p21 KD and *p* = 0.063 for Msi-1/p21 KD compared to controls). **2D**: Metabolic activity of Musashi-1 knockdown cells compared to control cells, measured by MTT assay (**D1** Ishikawa *p* = 0.003, *n* = 5; **D2** KLE *p* = 0.002, *n* = 4)

To investigate Msi-1-mediated proliferation changes, we then conducted colony formation assays. After knockdown using two siRNAs, Msi-1 depleted cells were 43% (KLE) and 45% (Ishikawa) less likely to form colonies compared to controls (Fig. 2A, Ishikawa: *p* < 0.001, *n* = 14; KLE: *p* = 0.027, *n* = 20; representative colonies in Fig. 2B). The effect was verified using siPOOLs, and similar results were observed (Fig. 2C1, C2, Ishikawa: *p* = 0.003, *n* = 17; KLE: *p* = 0.003, *n* = 8 for Msi-1 KD).

Similarly, in MTT assays, cell metabolism was reduced to about 80% (Ishikawa 81%, KLE 79%) of control cell levels after Msi-1 knockdown with two siRNAs (Fig. 2D, Ishikawa: *p* = 0.003, *n* = 5; KLE: *p* = 0.002, *n* = 4).

### Musashi-1 downregulation is associated with changes in cell cycle, cancer stem cell and proliferation genes

**Fig. 3:**
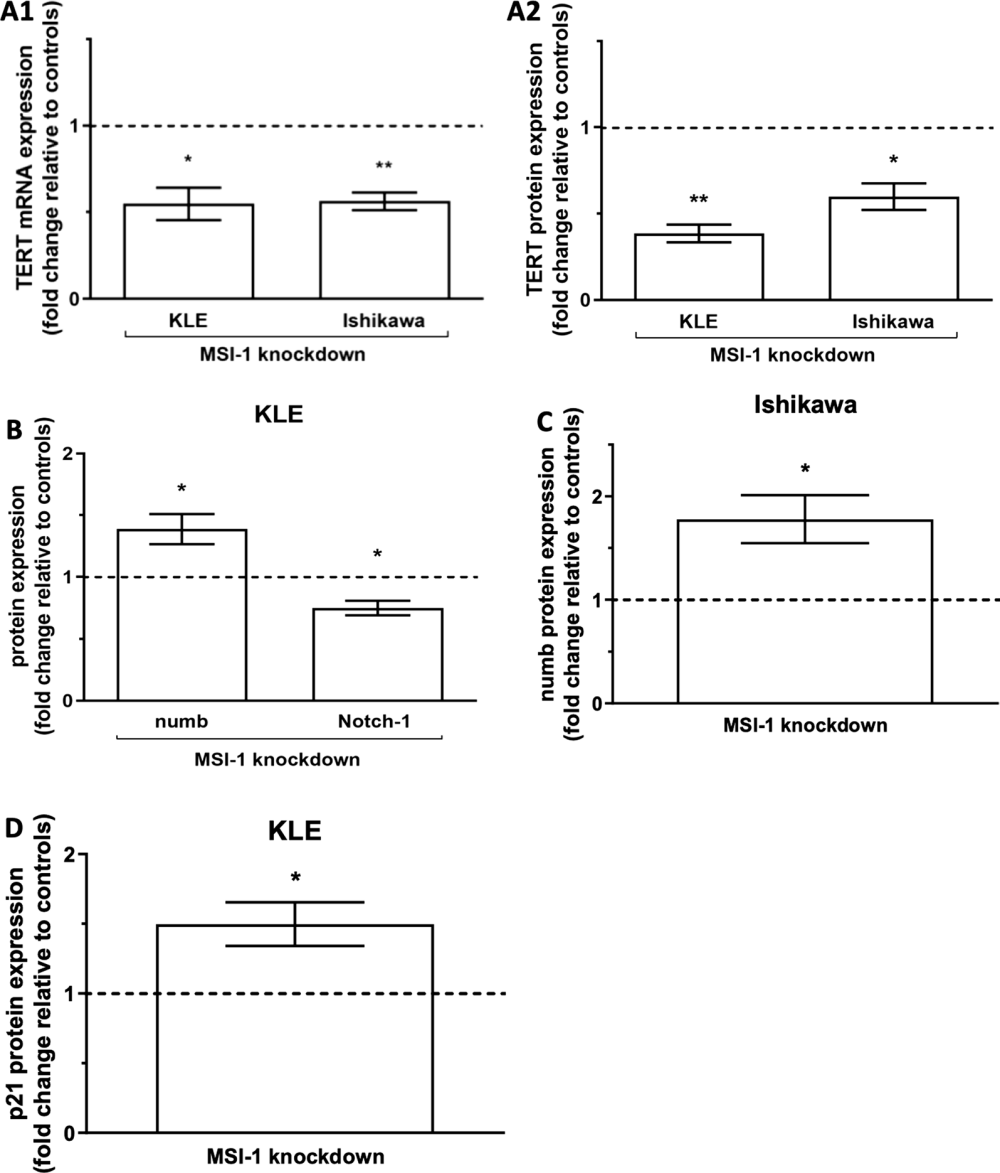
Musashi-1 downregulation is associated with changes in cell cycle, cancer stem cell and proliferation genes. **A** TERT (telomerase reverse transcriptase) expression is downregulated on both, mRNA (A1, Ishikawa *p* = 0.003 (mean delta ct values 20.27 for ctrl, 21.12 for Msi-1 depleted cells); KLE *p* = 0.018 (mean delta ct values 21.35 for ctrl, 22.49 for Msi-1 depleted cells); both *n* = 4) and protein (A2, Ishikawa *p* = 0.014 (relative protein expression level normalized to tubulin 0.23 for ctrl and 0.13 for Msi-1 KD), KLE *p* = 0.001 (relative protein expression level normalized to tubulin 5.71 for ctrl and 2.20 for Msi-1 KD); both *n* = 4) level in both cell lines. **B** Increased numb (*p* = 0.047, *n* = 4, relative protein expression level normalized to tubulin 4.50 for ctrl and 6.18 for Msi-1 KD) and decreased Notch-1 protein expression (*p* = 0.049, *n* = 3, relative protein expression level normalized to tubulin 0.14 for ctrl and 0.10 for Msi-1 KD) in the KLE cell line after Msi-1 knockdown. **C** Numb protein expression in Ishikawa cells is increased after Msi-1 silencing (*p* = 0.044, *n* = 4, relative protein expression level normalized to tubulin 1.87 for ctrl and 3.26 for Msi-1 KD). **D** Expression of p21 in KLE after Msi-1 knockdown (*p* = 0.048, *n* = 4, relative protein expression level normalized to tubulin 2.36 for ctrl and 3.98 for Msi-1 KD). Representative Western Blot signals are shown in Additional file 1: Fig. S4

TERT (Telomerase reverse transcriptase), the catalytic subunit of telomerase, was previously linked to Msi-1 expression [16]. After Msi-1 knockdown, TERT was downregulated on mRNA and protein levels in both cell lines (Fig. 3A, mRNA: *p* = 0.018, *n* = 4 for KLE and *p* = 0.003, *n* = 4 for Ishikawa; Western Blot p = 0.001, *n* = 4 for KLE and *p* = 0.014, *n* = 4 for Ishikawa, representative blots in Additional file 1: Fig. S4), a result supported by siPOOL Western Blot findings (*p* = 0.099, *n* = 2 for KLE and *p* = 0.046, *n* = 2 for Ishikawa, Additional file 1: Fig. S2).

Msi-1 plays an important role in activating the Notch pathway by targeting Notch-inhibitor numb [10]. Given that TCGA database analyses for this study identified Notch-1 as a negative prognostic marker in endometrial carcinoma (Additional file 1: Fig. S5, *p* = 0.004), we tried to characterize the relationship between Msi-1 and Notch-1. We found increased numb expression after Msi-1 knockdown in both cell lines (Fig. 3B, C, *p* = 0.047, *n* = 4 for KLE; *p* = 0.044, *n* = 4 for Ishikawa). Similar to exploratory findings in Ishikawa [17], Notch-1 was also decreased in KLE (Fig. 3B, *p* = 0.049, *n* = 3).

Silencing of Msi-1 has also been described to result in increased expression of p21, an anti-proliferative marker, in type 1 EC Ishikawa cells [17]. The same was true in type 2 KLE cells for both the approach involving two siRNAs (Fig. 3D, *p* = 0.048, *n* = 4) and the siPOOL approach (Additional file 1: Fig. 2, *p* = 0.054, *n* = 2). Given the importance of p21 for cell cycle arrest [29], we attempted to rescue proliferative function in Msi-1 knockdown cells by additionally targeting p21 using a dedicated p21 siPOOL. Knockdown was verified by Western Blot (Additional file 1: Fig. 2). We found that colony formation was significantly increased/rescued after additional p21 targeting in Msi-1 knockdown cells compared to Msi-1 knockdown alone in both cell lines (Fig. 2C, Ishikawa: *p* = 0.041, *n* = 17 for Msi-1 and *n* = 15 for Msi-1/p21 KD; KLE: *p* = 0.036, *n* = 8 for Msi-1 KD and *n* = 11 for Msi-1/p21 KD). Meanwhile, compared to control siRNA transfected cells, proliferation after Msi-1 and p21 double knockdown was slightly lower, but only marginally so (Fig. 2C, Ishikawa: *p* = 0.076, *n* = 17 for ctrl and *n* = 15 for Msi-1/p21 KD; KLE: *p* = 0.063, *n* = 9 for ctrl and *n* = 11 for Msi-1/p21 KD).

### Radioresistance and DNA-PKcs expression are decreased after Musashi-1 knockdown, while Musashi-1 expression is induced by irradiation

**Fig. 4:**
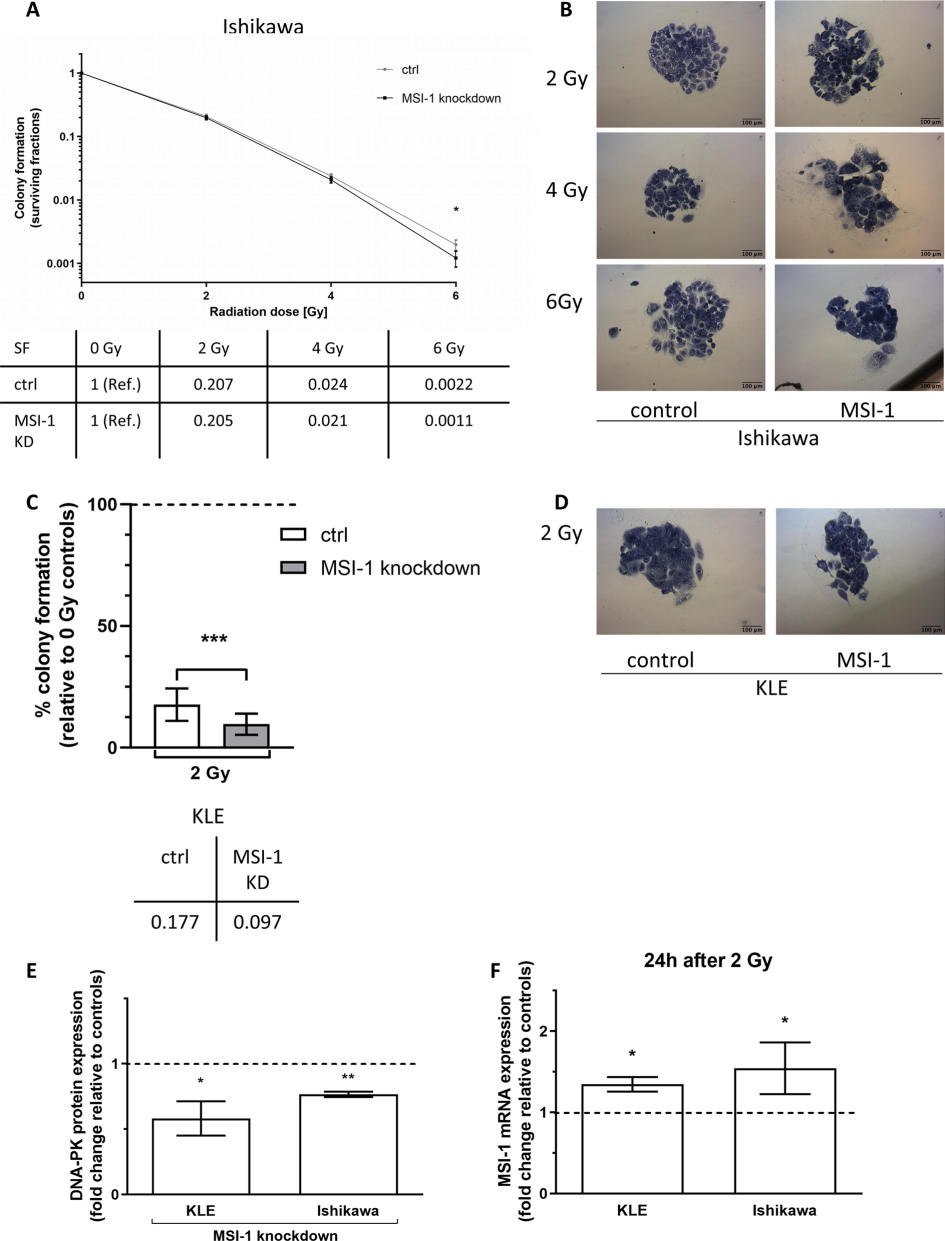
Radioresistance and DNA-PKcs expression are decreased after Musashi-1 knockdown, while Musashi-1 expression is induced by irradiation. **A**, **C** Surviving fractions of cells after irradiation in Ishikawa (**A**: 2 Gy and 4 Gy n.s., 6 Gy *p* = 0.046, *n* = 14, survival fractions (SF) presented in graph and corresponding table) and KLE cells (C: 2 Gy *p* = 0.0002, *n* = 18; SF presented in graph and corresponding table). Note logarithmic scale of y-axis. **B**, **D** Representative images of cancer cell colonies. **E** Protein expression of DNA-PKcs after Msi-1 knockdown (*p* = 0.008, *n* = 3 for Ishikawa (relative protein expression level normalized to tubulin 1.36 for ctrl and 1.05 for Msi-1 KD); *p* = 0.049, *n* = 4 for KLE (relative protein expression level normalized to tubulin 1.73 for ctrl and 1.16 for Msi-1 KD)). Representative Western Blot signals in Additional file 1: Fig. S4. **F** Msi-1 mRNA expression in wild-type cells 24 h after irradiation compared to unirradiated cells (*p* = 0.032 for KLE (mean delta ct values 17.20 for 0 Gy ctrl, 16.78 for 2 Gy irradiated cells), *p* = 0.042 for Ishikawa (mean delta ct values 16.66 for 0 Gy ctrl, 16.06 for 2 Gy irradiated cells); both *n* = 4)

Msi-1 knockdown sensitized cells to irradiation in both EC cell lines. However, radioresistance differed considerably: Ishikawa cells proved radioresistant at doses of 2, 4 and 6 Gy, while KLE cells did not form any colonies after doses of more than 2 Gy, precluding experiments involving higher doses. In Ishikawa, Msi-1 knockdown cells were increasingly more radiosensitive across doses of 2 and 4 Gy, finally showing a significant difference compared to controls after a 6 Gy dose (Fig. 4A, *p* = 0.046, *n* = 14, mean survival fraction after 2, 4 and 6 Gy presented in table). Meanwhile, in KLE, a significant radiosensitization after 2 Gy was observed (Fig. 4C, *p* = 0.0002, *n* = 18, mean survival fraction after 2 Gy presented in table). Representative pictures of colonies after different irradiation doses can be found in Fig. 4B for Ishikawa and 4D for KLE. Both results were additionally independently confirmed using siPOOL approaches (Additional file 1: Fig. S6; Ishikawa: *p* = 0.002, *n* = 13 for Msi-1 KD; KLE: *p* = 0.036, *n* = 5 for Msi-1 KD).

DNA-dependent protein kinase (DNA-PK) is critical for non-homologous end joining (NHEJ), the main repair mechanism of DNA double-strand breaks (DSBs) caused by ionizing radiation [30]. Expression of DNA-PKcs, the catalytic subunit of DNA-PK, was significantly decreased in both cell lines after Msi-1 knockdown (Fig. 4E, *p* = 0.008, *n* = 3 for Ishikawa and *p* = 0.049, *n* = 4 for KLE).

We then determined Msi-1 mRNA expression 24 h after irradiation with 2 Gy compared to unirradiated cells in wild-type Ishikawa and KLE cells. Msi-1 expression was significantly induced after treatment with 2 Gy in both cell lines (Fig. 4F, *p* = 0.032, *n* = 4 for KLE; *p* = 0.042, *n* = 4 for Ishikawa).

Finally, MTT assay after paclitaxel treatment did not reveal additional chemosensitization in both cell lines after Msi-1 silencing (Additional file 1: Fig. S7).

### Altered gene expression findings after Musashi-1 knockdown are underlined by RNAseq assays from human specimens

**Fig. 5:**
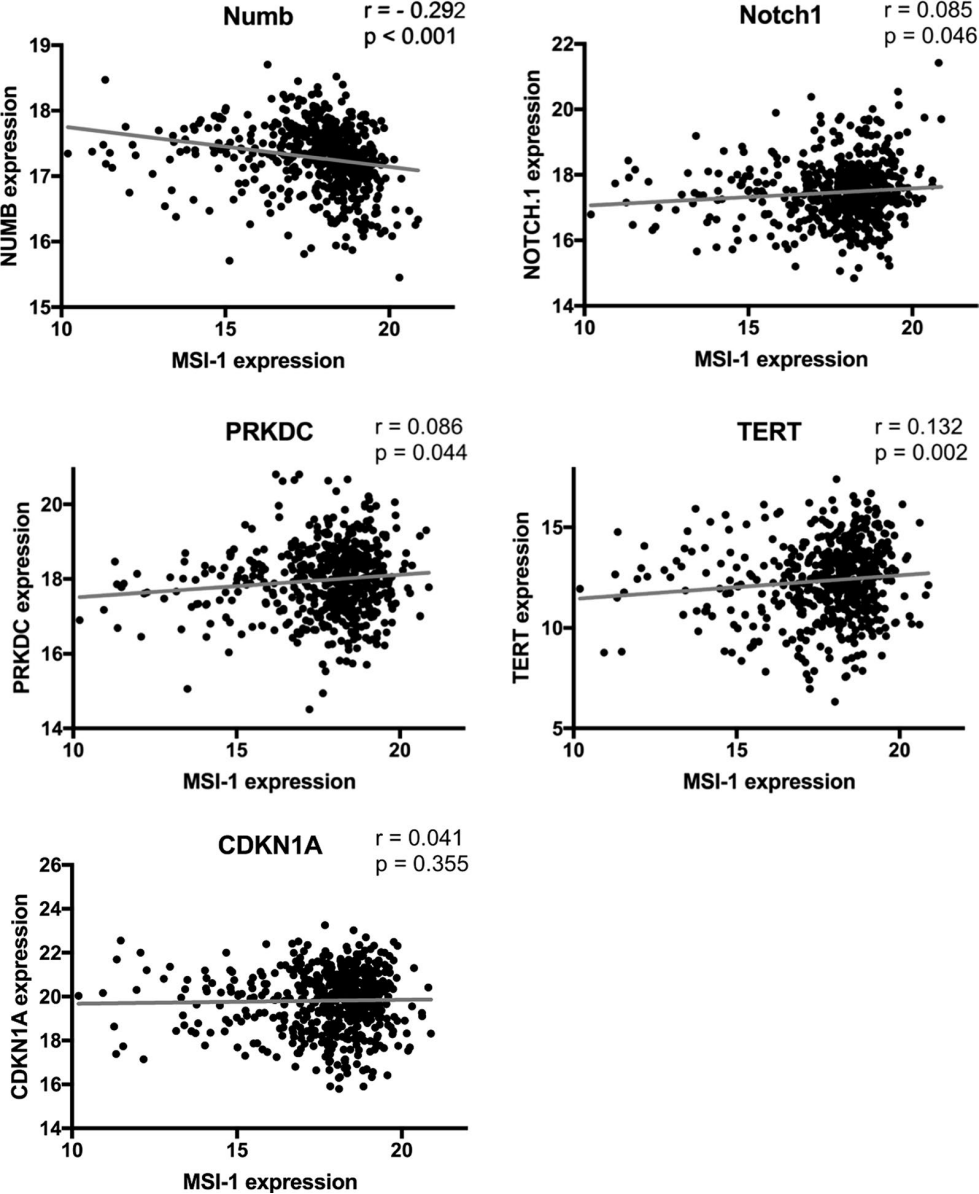
Gene expression correlations using TCGA database endometrial cancer RNA sequencing. Msi-1 expression is correlated with numb, Notch-1, PRKDC (DNA-PKcs), TERT and CDKN1A (p21). Spearman’s r and respective p values are presented

We then aimed to validate altered expression of Msi-1 targets in vivo. We correlated the expression of the differentially expressed genes identified in this study to Msi-1 expression in primary patient samples, again using the TCGA dataset. All target genes except for p21 (where no effect was seen) showed expression correlations similar to effects seen after Msi-1 knockdown in vitro (Fig. 5, *p* values presented in figure).

### Musashi-1 downregulation limits tumor growth in vivo

Finally, we validated our strong anti-proliferative in vitro findings with a xenograft in vivo model. We injected Ishikawa cells into immunodeficient nude mice, followed by regular Msi-1 or control siRNA injections.

**Fig. 6:**
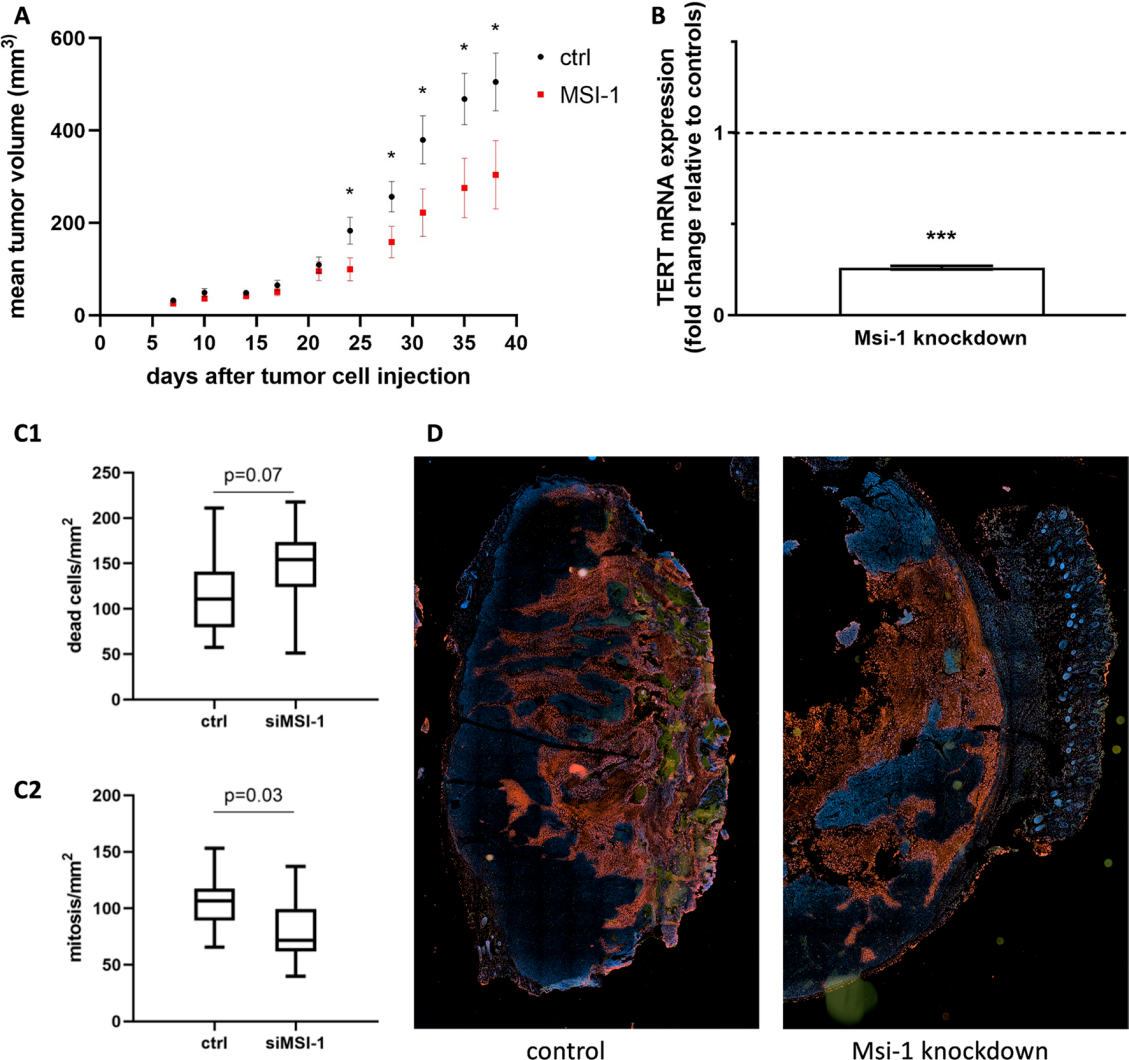
In vivo Musashi-1 downregulation limits tumor growth. **A** Xenograft tumor growth over time in Msi-1-siRNA and control siRNA-treated mice (*p* < 0.05 after day 24: *p* = 0.038 at d (day) 24, *p* = 0.048 at d 28, *p* = 0.041 at d 31, *p* = 0.032 at d 35, *p* = 0.047 at d 38, *n* = 15). **B** TERT (telomerase) mRNA expression in Msi-1 depleted tumors compared to controls (*p* = 0.001, *n* = 27 for ctrl (mean delta ct value 8.43), *n* = 21 for Msi-1 KD (mean delta ct value 10.37)). **C** Dead cell (**C1**) and mitotic cell (**C2**) area in Msi-1 depleted tumors compared to controls (*n* = 14). **D** Representative pictures of control (left) and Msi-1 depleted (right) tumor microsections after immunofluorescence staining (DNA = blue, mitotic activity = green, dead cells = red)

For the first three weeks, growth rates were largely similar between groups. However, starting after day 25, tumor volumes were significantly increased in the control group (Fig. 6A, *p* = 0.038 at d (day) 24, *p* = 0.048 at d 28, *p* = 0.041 at d 31, *p* = 0.032 at d 35, *p* = 0.047 at d 38, *n* = 15). Mean tumor volume on day 7 was 25.69 mm^3^ for Msi-1-depleted tumors and 32.22 mm^3^ for the control group. In the final measurement on day 38, Msi-1 tumors had grown to a mean volume of 304.29 mm^3^ compared to control tumors 505.37 mm^3^. In five mice, only miniscule tumor masses were detected, four in Musashi-1 knockdown and one in the control group. Measurements for these tumors ranged from 7.02 to 52 mm^3^ on day 38. For these tumors, no RNA isolation and therefore no further qPCR analyses were feasible.

Immunohistochemical staining after tumor explantation revealed more apoptotic cells in Msi-1 knockdown tumors, while the mitotic count was significantly decreased (Fig. 6C, *p* = 0.071 for dead cells, *p* = 0.026 for mitosis, *n* = 14).

We confirmed Msi-1 knockdown using RT-qPCR analyses on tumor tissues (Additional file 1: Fig. S1). Additionally, paraffin-embedded sections were stained with a Msi-1 antibody. Microscopy illustrated high Msi-1 expression in control tumors, while staining was strongly reduced in Msi-1 knockdown tissues (Additional file 1: Fig. S1).

Finally, in accordance with our in vitro studies, TERT (telomerase) mRNA expression was strongly downregulated in Msi-1 depleted tumors (Fig. 6B, *p* = 0.001, *n* = 27 for ctrl and *n* = 21 for Msi-1 KD).

## Discussion

**Fig. 7:**
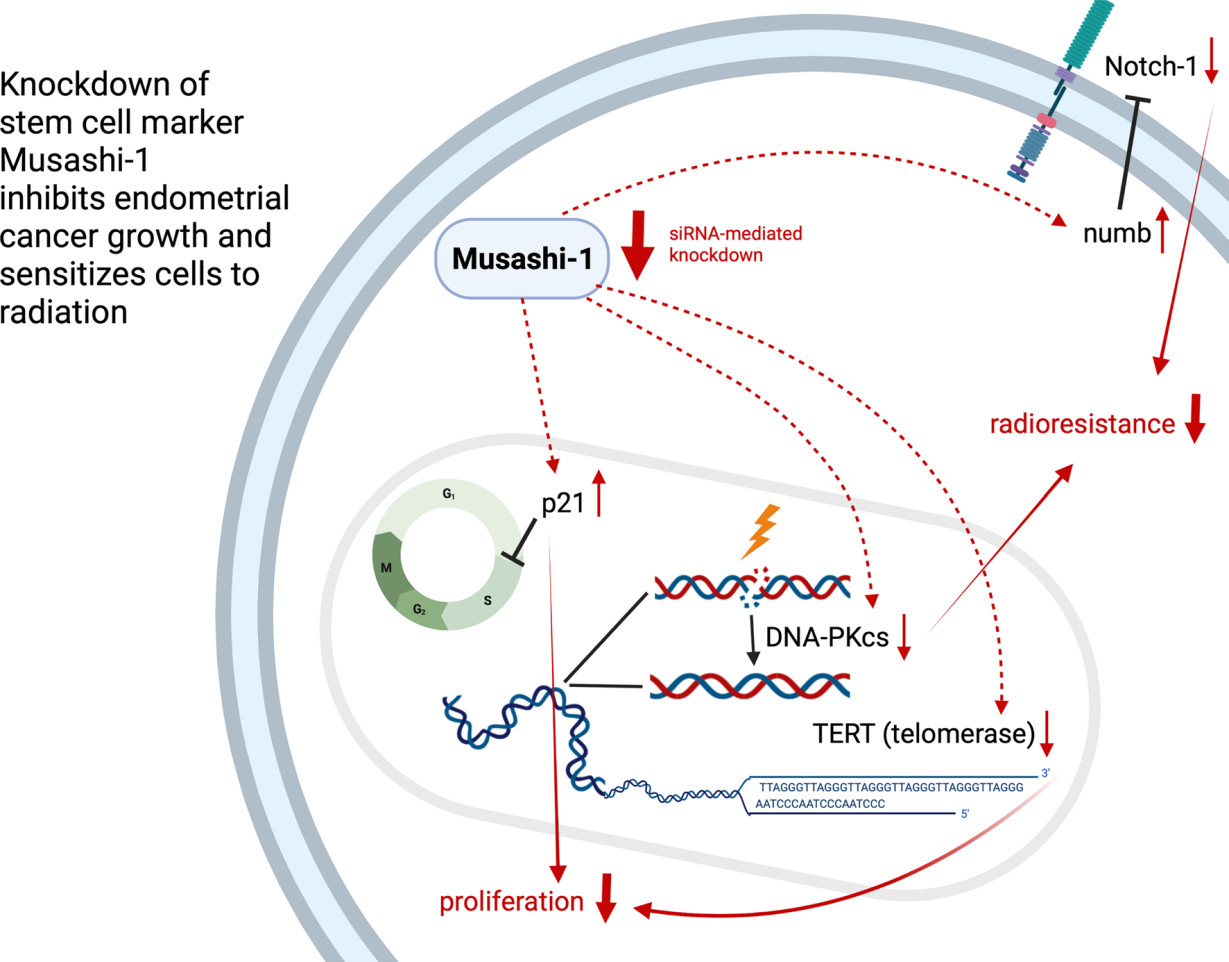
Graphical abstract of Msi-1-mediated changes in gene expression and cell behavior. The graph was created with Biorender.com

Here, we characterized the therapeutic potential of Msi-1 targeting. Focusing on cell proliferation and therapy resistance, we aimed to understand whether artificial Msi-1 knockdown may be a therapeutic venue to impede tumor growth and increase chemo- and radiosensitivity. Mechanistic findings discussed below are summarized in Fig. 7.

Msi-1 has previously been demonstrated to be overexpressed in a wide variety of malignancies, including in brain [12, 31], gastrointestinal [13, 32] and other gynecologic cancers [15, 33, 34]. A previous study by our group found Msi-1 highly expressed in a small cohort (14 patients) in EC [16]. Results from another 50 patient cohort were in agreement [35]. Our recent analyses confirmed these findings in more than 500 primary cancer tissues of the TCGA database and verified them independently in the CPTAC database. We also found a lower level of promoter methylation in cancer tissues, another indicator of increased Msi-1 expression. Msi-1 expression was more than doubled compared to normal endometrial tissues in TCGA-based analyses, encouraging subsequent Msi-1 knockdown investigations.

Clonogenic capability and cell metabolism were strongly reduced in both cell lines after Msi-1 knockdown. Our in vitro findings were validated by the in vivo xenograft study, confirming significantly slowed growth in Msi-1 knockdown tumors. Effects in vivo and in vitro consistently point toward a 40% decrease in proliferation. Additional experiments using a Msi-1-specific siPOOL further confirmed previously performed in vitro experiments. Our findings are consistent with previous studies in ovarian cancer [36], glioblastoma [37] and our previous study in breast cancer [38].

In our xenograft study, the size difference became apparent in the exponential growth phase of the tumors. Hypoxia is the main challenge in early tumor growth [39] and likely obscured any early growth differences as it may have affected Msi-1 knockdown and control tumors both. In five mice, only small tumor masses were detected, four in Msi-1 knockdown and one in control group. This shows that in some cases, Msi-1 knockdown may have been able to halt tumor growth completely in early stages.

Possible mechanistic pathways include cell cycle arrest previously associated with Msi-1 knockdown [17], knockdown-dependent upregulation of the anti-proliferative marker p21 [40], as well as downregulation of Notch [41] and TERT [42], both of which have been implicated in cell proliferation.

The anti-proliferative marker p21 is a cyclin-dependent kinase inhibitor, an important regulatory protein in cellular processes like differentiation, apoptosis, stem cell self-renewal and metastasis. P21 mostly acts as a tumor suppressor, but has also been linked to oncogenesis [40]. As a translational repressor of p21 during neural development [43], Msi-1 also targets p21 in malignances including cervical carcinoma [44] and breast cancer [15]. Surprisingly*, *in vivo database analyses showed no significant association between p21 and Msi-1. However, in our prior exploratory analyses we found p21 to be upregulated in Ishikawa cells after Msi-1 knockdown [17]. Here, we confirmed upregulation of p21 in KLE, a type 2 EC cell line, solidifying the antagonistic relationship. Given p21’s well-described role as an anti-proliferative marker [40], we aimed to evaluate its role for cell proliferation changes in the Musashi regulatory network. We hypothesized that experimental p21 silencing may be able to alleviate the loss of proliferative function after Msi-1 knockdown. We found that additional p21 targeting after Msi-1 knockdown increased colony formation compared to Msi-1 knockdown alone, demonstrating the importance of the Msi-1-p21-axis for proliferative function in the Musashi regulatory network. However, proliferative function was not entirely returned to normal, pointing to additional factors and Musashi-related pathways that are likely involved in anti-proliferative signaling, such as loss of cancer stem cell characteristics after Msi-1 knockdown.

Cancer stem cells (CSCs) are thought to be essential for development, metastasis and recurrence of cancer as well as chemotherapy and radiation resistance in endometrial cancer [45, 46]. Msi-1 has previously been identified as a CSC marker in EC [17], as well as in other malignancies [15, 47, 48]. In the present study, we identify CSC genes associated with Msi-1 expression: TERT and the numb/Notch pathway.

TERT is the catalytic subunit of telomerase, known to be essential for tumor cells to reach unlimited replication potential. Physiologically expressed in gametes and stem cells [49, 50], it is also known to be a key CSC marker [51]. Our group has previously demonstrated that TERT and Msi-1 are co-localized [16]. In the present study, we extend these findings: In vitro, we found TERT to be downregulated after Msi-1 knockdown. In vivo, TERT was associated with Msi-1 expression in TCGA patient samples and downregulated in our Msi-1 knockdown xenograft mouse model. Many studies have focused on telomerase inhibition given its therapeutic potential. However, currently, there are no clinically approved options for targeting telomerase due to mutations and complexity of the enzyme [52, 53]. Indirect targeting of telomerase via Msi-1 inhibition might be a potential therapeutic option.

Msi-1 is known to be a direct inhibitor of numb [10], a well-known inhibitor of the Notch pathway. Thus, ultimately, Msi-1 expression supports Notch pathway activation [10, 54]. Notch signaling has been connected to therapy resistance and CSCs, including in EC [16, 17, 55]. Here, we demonstrate that numb is upregulated in both cell lines after Msi-1 knockdown. Meanwhile, Notch-1 is downregulated in KLE. Similar results for Ishikawa have been demonstrated in our exploratory study [17]. These in vitro findings are supported by our TCGA database in vivo data. Lastly, we show that high Notch-1 expression is associated with decreased survival in the TCGA database, underlining the therapeutic relevance of the Msi-1-Notch-1 axis.

CSCs have been demonstrated to be key obstacles to conventional therapy [56], including irradiation [57]. Given the subsequent loss of CSC characteristics, we hypothesized that Msi-1 knockdown might sensitize cells to irradiation, which was confirmed during our experiments. While both cell lines demonstrated some degree of radiosensitization after Msi-1 knockdown, there were some differences regarding radiation response: KLE, known to be a radiosensitive cell line [58], lost colony forming ability completely after doses higher than 2 Gy. Meanwhile, Ishikawa cells formed colonies after all applied doses. Both cell lines showed Msi-1 knockdown-related radiosensitization effects, though effects were more pronounced in KLE than in Ishikawa cells. Msi-1 has been attributed radiosensitizing effects before in multiple tumor entities [12, 13, 15, 34, 59].

While cancer stem cells have generally been associated with radioresistance, there is also specific evidence for the involvement of both TERT and the Notch pathway in radiation response: Loss of TERT is known to sensitize cancer cells to both irradiation and chemotherapy [60] and specifically impairs DNA damage repair [61], a key resistance mechanism after ionizing irradiation. Radioresistance via Notch-1 was reported for malignant gliomas [62] and numb/notch-1 signal pathway inhibition enhanced radiosensitivity in lung cancer [63], melanoma [64] and pancreatic cancer [65]. Decreased radioresistance in both cell lines is therefore understood to be partly caused by decreased Notch-1 and TERT after Msi-1 knockdown.

Additionally, DNA-PKcs has an essential role in DNA repair after radiation-induced double-strand breaks [30]. Downregulation of DNA-PKcs, found here after Msi-1 knockdown in vitro and in vivo as a database correlation with low MSI-1 expression, may be responsible for the decrease in radioresistance. This effect has previously been seen in glioblastoma [12] and breast cancer [38]. Finally, Msi-1 expression was induced after irradiation in wild-type cells, underlining Msi-1’s importance for radioresistance. This is in line with findings in glioblastoma cells [12].

While we saw a strong decline in metabolic cell activity after Msi-1 knockdown alone, addition of paclitaxel did not result in further eradication of Msi-1 knockdown cells compared to controls. This indicates that there is no synergism between treatments. However, both interventions largely seem to have additive character. In KLE, at two high concentrations of chemotherapy, Msi-1 knockdown cells displayed a slightly higher cell metabolism, meaning effects may not be entirely additive at all concentrations. These findings are in contrast to previous studies in ovarian cancer, where Musashi-1 and Musashi-2 were described to be regulators of paclitaxel sensitivity [34, 36, 66].

Our general finding that Msi-1 knockdown sensitizes cancer cells to therapy and limits proliferation indicates therapeutic potential. It is in agreement with (and extends beyond) the limited number of previous studies evaluating the role of Musashi-1 in endometrial cancer: Previous work showed that Musashi-1 is overexpressed in endometrial cancer [16] and high expression is prognostically unfavorable [18]. Msi-1 has also been demonstrated to be overexpressed in endometrial stem cells [35] and to be associated with stem cell pathways both in endometrial cancer [17] and endometriosis [67]. Finally, Musashi-1 has been identified as a potential therapeutic target in other malignancies, including breast cancer [15] and glioblastoma [68] and here, we report an in-depth evaluation of its targeting in endometrial cancer.

There are some notable limitations to this study. First, radiation and chemotherapy treatment experiments were only performed in vitro, not in vivo. Second, radiation experiments in KLE cells were only performed for a 2 Gy dose. However, no colonies were seen at higher doses, precluding 4 and 6 Gy analyses.

## Conclusions

In the present study, we show decreased cell proliferation and radioresistance after siRNA-mediated Msi-1 knockdown in two endometrial carcinoma cell lines. In vitro and in patient database analyses, we demonstrate that low Msi-1 is linked to a decrease in Notch-1, DNA-PKcs and TERT, all related to cancer stem cells and therapy resistance, and an increase in p21, an anti-proliferative marker. Finally, xenograft mouse models allowed validation of anti-proliferative Msi-1 knockdown effects in vivo. Taken together, our findings indicate that Msi-1 targeting is a therapeutically interesting option to decrease endometrial cancer cell proliferation and radioresistance. Future trials should include in vivo assessments of therapy resistance after Msi-1 knockdown, use of CRISPR to evaluate Msi-1 knockout as opposed to siRNA-based knockdown, evaluation of Msi-1 and Msi-2 double knockdown considering close similarities between both proteins as well as translational assessment of Musashi inhibitors given potential clinical value.

## Supplementary Information

**Additional file 1. Fig. S1**: Musashi-1 knockdown verification. **Fig. S2**: siPOOL knockdown verification (Musashi-1 and p21). **Fig. S3**: Representative Western blots for siPOOL experiments. **Fig. S4**: Representative Western blots for siRNA experiments. **Fig. S5** Notch-1 expression and endometrial cancer patient survival. **Fig. S6**: Irradiation experiments after Musashi-1 siPOOL knockdown. **Fig. S7**: MTT assay after paclitaxel treatment. **Table S1**: Msi-1 knockdown siRNAs/siPOOLs. **Table S2**: qPCR TaqMan probes. **Table S3.1** and **S3.2**: western blotting antibodies.

## Data Availability

All data generated or analyzed during this study are included in this published article.

## Abbreviations

CPTAC: The National Cancer Institute’s Clinical Proteomic Tumor Analysis Consortium
CSC: Cancer stem cell
ctr: Control
d: Day
DAPI: 4′,6-Diamidino-2-phenylindol
DSBs: Double-strand breaks
DNA-PKcs: DNA protein kinase catalytic subunit
DMSO: Dimethyl sulfoxide
EC: Endometrial carcinoma
FCS: Fetal calf serum
IR: Ionizing radiation
KD: Knockdown
Msi-1: Musashi RNA-binding protein 1
MTT: 3-(4,5-Dimethylthiazol-2-yl)-2,5-diphenyltetrazolium bromide
NHEJ: Non homologous end joining
qPCR: Quantitative polymerase chain reaction
RNAseq: RNA sequencing
RT-qPCR: Quantitative reverse transcription polymerase chain reaction
SDS: Sodium dodecyl sulfate
s.e.m.: Standard error of the mean
siRNA: Small interfering RNA
TCGA: The Cancer Genome Atlas
TERT: Telomerase reverse transcriptase
tpm: Transcripts per million
UALCAN: The University of Alabama Cancer Database
