# Supplementary material for: Knockdown of the stem cell marker Musashi-1 inhibits endometrial cancer growth and sensitizes cells to radiation
Source: Stem Cell Res Ther. 2022 May 26;13:212. doi: 10.1186/s13287-022-02891-3 (PMC9137084; doi:10.1186/s13287-022-02891-3)
Supplement: Supplementary file 1 — Additional file 1. Fig. S1: Musashi-1 knockdown verification. Fig. S2: siPOOL knockdown verification (Musashi-1 and p21). Fig. S3: Representative Western blots for siPOOL experiments. Fig. S4: Representative Western blots for siRNA experiments. Fig. S5 Notch-1 expression and endometrial cancer patient survival. Fig. S6: Irradiation experiments after Musashi-1 siPOOL knockdown. Fig. S7: MTT assay after paclitaxel treatment. Table S1: Msi-1 knockdown siRNAs/siPOOLs. Table S2: qPCR TaqMan probes. Table S3.1 and S3.2: western blotting antibodies. [file 13287_2022_2891_MOESM1_ESM.docx]

**Supplementary Materials**

**Supplementary Figure S1**

**A** **B**

**
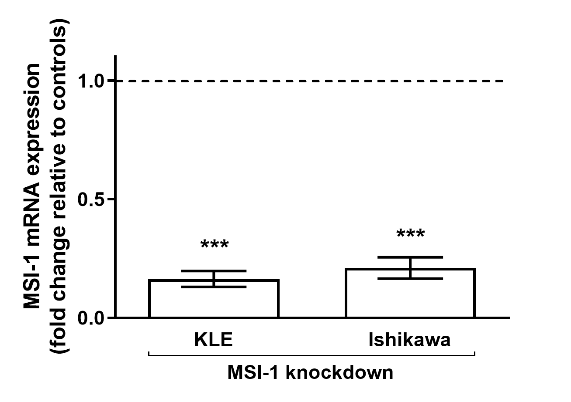
**
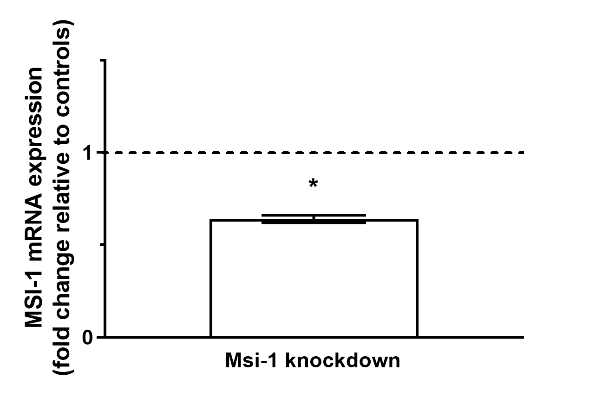


**C D**


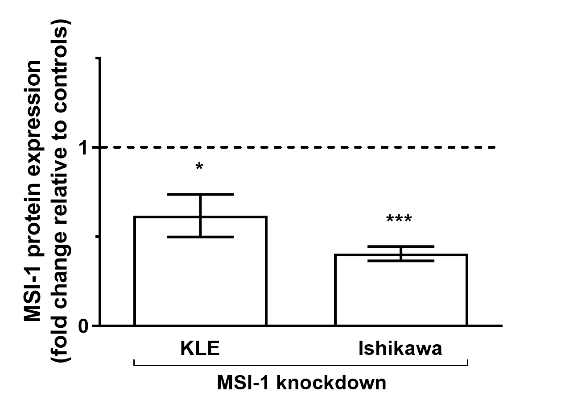


**E**

**F**

*
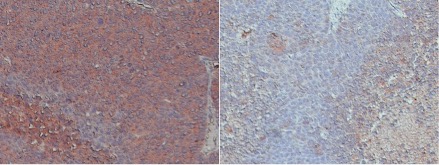
*

***
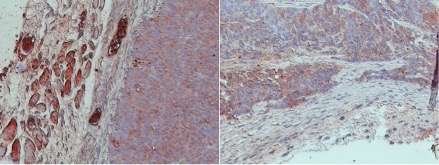
***

control Msi-1 knockdown

**Supplementary Figure S1**: Musashi-1 knockdown verification investigations. Musashi-1 knockdown was performed using an siRNA-based approach. Decreased Msi-1 mRNA levels after Msi-1 knockdown *in vitro* (**1A**, KLE p < 0.001 n = 5, mean delta ct values 16.6 for ctrl and 19.36 for Msi-1 depleted cells; Ishikawa p < 0.001 n = 4, mean delta ct values 16.14 for ctrl and 18.52 for Msi-1 depleted cells) and *in vivo* (**1B**, p = 0.018 n = 28 for ctrl (mean delta ct value 5.03), n = 22 for Msi-1 KD (mean delta ct value 5.67)). **1C**: *In vitro* knockdown was further evaluated via Western Blot analysis (KLE p = 0.049, n = 4); Ishikawa p = 0.001 n = 4). **1D**: Unenhanced original Western blot signals. 1**E & 1F**: Representative pictures of tumor microsections after staining with Msi-1 antibody to evaluate knockdown success (left: control group, right: Msi-1 knockdown). Both pictures were taken under 30x magnification. In **E** sections from tumor central area are presented, in **F** the periphery is shown.

**Supplementary Figure S2**

**A B**

**
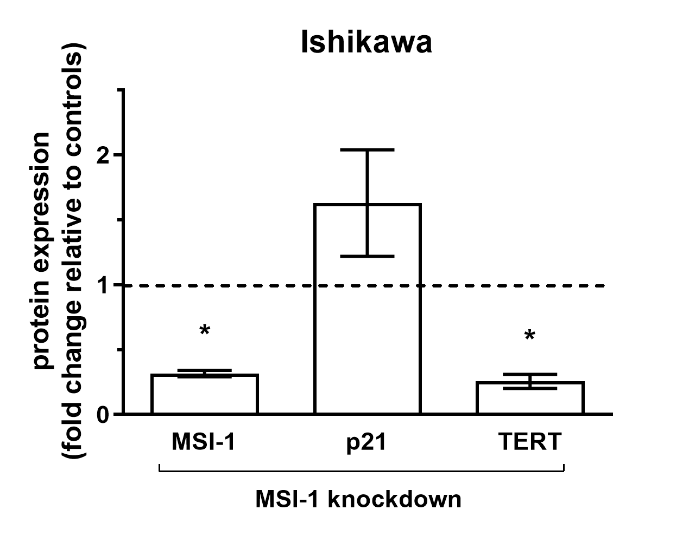

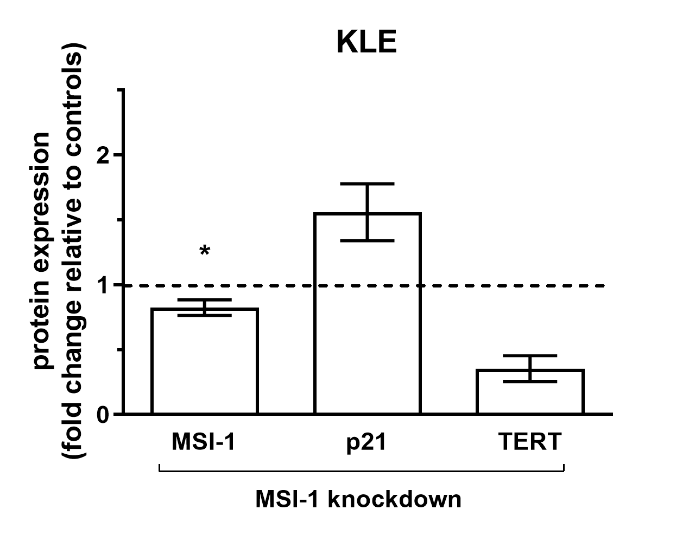
**

**C D**

**
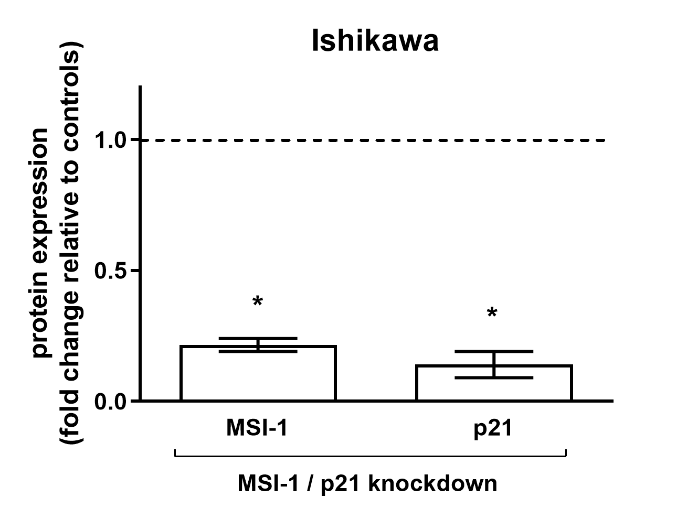

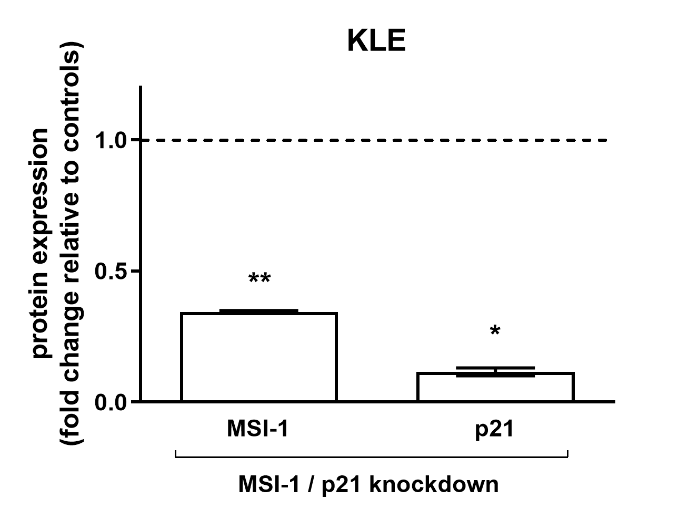
**

**Supplementary Figure S2:** Western blot results following siPOOL knockdown of Msi-1 (A&B) and Msi-1 and p21 (C&D). **2A & B**: Musashi-1 knockdown was verified via western blotting of reduced Msi-1 protein expression. Additionally, p21 was overexpressed while TERT was downregulated (**2A**: Ishikawa: n = 2, Msi-1: p = 0.021, p21: p = 0.366, TERT: p = 0.046; **2B**: KLE: n = 2, Msi-1: p = 0.039, p21: p = 0.054, TERT: p = 0.099). **2C & D**: Knockdown confirmation of Msi-1 and p21 in Ishikawa (**2A**, Msi-1: p = 0.022, p21: p = 0.038, n = 2) and KLE (**2B**, Msi-1: p = 0.007, p21: p = 0.011, n = 2). Representative western blot signals are presented in Supplementary Figure S3. Experiments were performed in duplicates only as they were solely designed to validate previously performed triplicate knockdown results after Msi-1 silencing with two siRNAs.

**Supplementary Figure S3**

**Supplementary Figure S3**: Unenhanced original Western Blot signals for presented analyses in Supplementary Figure S2.

**Supplementary Figure S4**

**Supplementary Figure S4**: Unenhanced original Western Blot signals for presented analyses in Figure 3 and 4: Decrease in DNA-PKcs, TERT and Notch-1 after MSI-1 knockdown and increase in numb are presented.

**Supplementary Figure S5**

**Supplementary Figure S5**: High expression of Notch-1, downstream target of Musashi-1, is associated with decreased overall survival in endometrial carcinoma patients (The Cancer Genome Atlas (TCGA) dataset, presented via the University of Alabama Cancer Database (UALCAN) web resource).

**Supplementary Figure S6**

**A B**

**
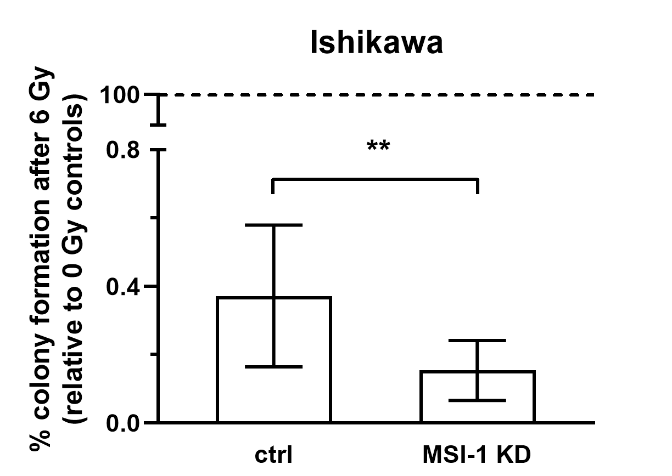

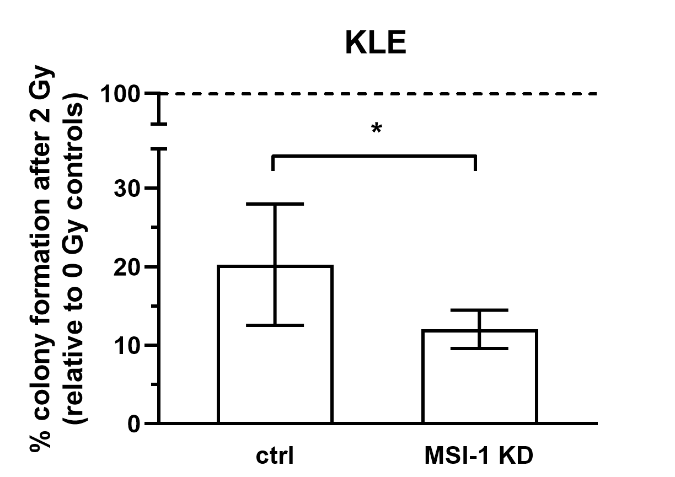
**

**Supplementary Figure S6**: Radioresistance decreases after Msi-1 KD. Please note the different scale on Y-axis. **6A**: Ishikawa: mean plating efficiency 0.004 for ctrl and 0.0015 for Msi-1 KD, p = 0.002, n= 13 for Msi-1 KD; **6B** KLE: mean plating efficiency 0.206 for ctrl and 0.120 for Msi-1 KD, p = 0.036, n = 5 for Msi-1 KD,

**Supplementary Figure S7**


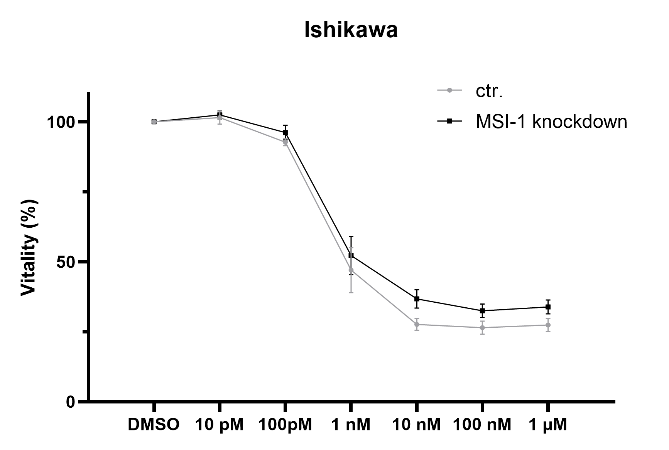


**Supplementary Figure S7**: Chemoresistance is largely unchanged after Msi-1 knockdown compared to controls. Cells were treated with paclitaxel for 96 hours. Afterwards, cell metabolism was measured using MTT assay (n.s. for Ishikawa; KLE n.s. for 10 pM, 100 pM, 1 nM, 10 nM, p = 0.010 for 100 nM and p = 0.024 for 1 µM; n = 6 for both cell lines).

**Supplementary Table S1**

siRNAs used for Musashi-1 knockdown.

|  | siRNA | Manufacturer | ID | Targeted sequence | Exons |
| --- | --- | --- | --- | --- | --- |
| In vitro | MSI-1 | Thermofisher Scientific | s8980 | GGUUCGGGUUUGUCACGUUtt | 2, 3, 4, 5, 7, 8 |
| In vitro | MSI-1 | Thermofisher Scientific | s8979 | GCAAUAUUUUGAGCAGUUUtt | 2, 3, 6 |
| In vivo | MSI-1 | Thermofisher Scientific | 143570 | GCUCGACUCCAAAACAAUU | 4, 5 |
| In vivo | MSI-1 | Thermofisher Scientific | 11591 | GGACGUGAAGCAAUAUUUU | 2, 3, 6 |
| Negative control | sinegctr | Thermofisher Scientific | 4390843 |  |  |

Negative control siRNA 4390843 was used for both *in vitro* and *in vivo* studies.

|  | siRNA | Manufacturer | Manufactuer ID | Transcripts targeted |
| --- | --- | --- | --- | --- |
| In vitro | MSI-1 | siTOOLs Biotech | siPOOL  NCBI Gene ID 4440 | **NM_002442, XM_006719403, XM_006719404, XM_011538361, XM_011538362, XM_011538363, XM_011538364, XM_011538365, XM_011538366, XM_011538368, XM_011538370, XM_011538371** |
| In vitro | p21 | siTOOLS Biotech | siPOOL (CDKN1A)-003  NCBI Gene ID 1026 | **NM_000389, NM_001220777, NM_001220778, NM_001291549,** **NM_078467** |
| Negative control |  | siTOOLS Biotech | Negative ctrl. siPOOL |  |

**Supplementary Table S2**

TaqMan probes used for qPCR experiments.

| **Gene** | **Manufacturer** | **Manufacturers ID** |  |
| --- | --- | --- | --- |
| 18S | Thermofisher Scientific | Hs99999901_m1 |  |
| MSI-1 | Thermofisher Scientific | Hs00159291_m1 | In vitro |
|  | Thermofisher Scientific | Hs01045894_m1 | In vivo |
| TERT (telomerase) | Thermofisher Scientific | Hs00162669_m1 | In vitro |
|  | Thermofisher Scientific | Hs00972656_m1 | In vivo |

**Supplementary Table S3.1 and S3.2**

Antibodies used for western blotting.

| **Target** | **Manufacturer** | **Catalogue number** | **Secondary antibody** |
| --- | --- | --- | --- |
| MSI-1 | Santa Cruz | sc-135721 | mouse |
| Numb | Santa Cruz | sc-136554 | mouse |
| Notch-1 | Santa Cruz | sc-376403 | mouse |
| α-tubulin | Santa Cruz | sc-5286 | mouse |
| DNA-PKcs | Santa Cruz | sc-5282 | mouse |
| Anti-telomerase (Ab-2) | Sigma-Aldrich | 582005 | rabbit |
| p21^WAF1/CIP1^ | Cell Signaling | 2947 | rabbit |

| **Secondary antibodies** | **Manufacturer** | **Manufacturers ID** |
| --- | --- | --- |
| Mouse IgG HRP-conjugated Antibody | R&D Systems | HAF007 |
| Rabbit IgG HRP-conjugated Antibody | R&D Systems | HAF008 |
